# Side effects and Immunogenicity following administration of the Sputnik V COVID-19 vaccine in health care workers in Iran

**DOI:** 10.1038/s41598-021-00963-7

**Published:** 2021-11-02

**Authors:** Farhang Babamahmoodi, Majid Saeedi, Reza Alizadeh-Navaei, Akbar Hedayatizadeh-Omran, Seyed Abbas Mousavi, Gasem Ovaise, Shirafkan Kordi, Zahra Akbari, Mazaher Azordeh, Fatemeh Ahangarkani, Ahmad Alikhani

**Affiliations:** 1 Antimicrobial Resistance Research Center, Communicable Diseases Institute, Mazandaran University of Medical Sciences, Sari, Iran; 2 Department of Pharmaceutics, Faculty of Pharmacy, Mazandaran University of Medical Sciences, Sari, Iran; 3 Gastrointestinal Cancer Research Center, Non-Communicable Diseases Institute, Mazandaran University of Medical Sciences, Sari, Iran; 4 Psychiatry and Behavioral Sciences Research Center, Addiction Institute, Department of Psychiatry, Mazandaran University of Medical Sciences, Sari, Iran; 5 Mazandaran University of Medical Sciences, Sari, Iran

**Keywords:** Diseases, Health care, Medical research

## Abstract

The Sputnik V is a COVID- 19 vaccine developed by the Gamalia institute of epidemiology and microbiology and released on August 11, 2020. We provided independent evidence on side effects and immunogenicity following the administration of the Sputnik V COVID-19 in Iran. In this observational study, the healthcare workers who were vaccinated with the Sputnik V COVID-19 vaccine within February and April 2021 were evaluated. Among a total of 13,435 vaccinated healthcare workers, we received 3236 self-declaration reports of Sputnik V associated adverse events with the mean age 39.32 ± 10.19 years old which 38.8% were men and 61.2% were women. Totally 68.8% of females versus 66.2% of males reported side effects after receiving the first dose and 31.2% of females versus 33.8% of males reported side effects after the second dose of vaccine. The most common side effect was a pain in the injection site (56.9%), fatigue (50.9%), body pain (43.9%), headache (35.7%), fever (32.9%), joint pain (30.3%), chilling (29.8%) and drowsiness (20.3%). Side effects of the vaccine were significantly more frequent in females and younger individuals. Among a total of 238 participants, more than 90% after the first and second dose of vaccine had a detectable level of SARS-CoV-2 RBD antibody and SARS-CoV-2 neutralizing antibody. Although the overall rate of adverse effects was higher than the interim results from randomized controlled trials, our findings support the manufacturer’s reports about the high humoral immunogenicity of vaccine against COVID-19.

## Introduction

The COVID-19 pandemic has resulted in more than 226,844,344 confirmed cases and more than 4,666,334 deaths (17 September 2021) worldwide^1^. In February 2020, Iran appeared as one of the first countries of the COVID-19 epidemic, and the number of infected people quickly exceeded the level of infection in other countries at that time. The clinical spectrum of the disease varies from asymptomatic or mild to severe cases leading to acute respiratory syndrome and respiratory failure and death. To date, there is no conclusive evidence for the effectiveness of current antiviral therapies. Global efforts are focused on producing safe and effective vaccines to prevent COVID-19, and there are currently several temporarily licensed vaccines against COVID-19 in the world. As of June 2021, 18 vaccines including two mRNA vaccines (the Pfizer–BioNTech vaccine and the Moderna vaccine), five conventional inactivated vaccines (BBIBP-CorV, CoronaVac, Covaxin, WIBP-CorV and CoviVac), four viral vector vaccines (Sputnik V, the Oxford–AstraZeneca vaccine, Convidecia, and the Johnson & Johnson vaccine), and two protein subunit vaccines (EpiVacCorona and RBD-Dimer) approved by at least one national regulatory authority for public use in countries. Due to the limited number of available vaccines, many countries have started vaccine distribution programs, prioritizing those most at risk and transmitting the disease, such as health care workers. In Iran, COVID-19 vaccination was started on February 9, 2021, in the first phase for health care workers. The first COVID-19 vaccination in Iran performed using Sputnik V. Sputnik V is a COVID- 19 vaccine developed by the Gamalia institute of epidemiology and microbiology and released on August 11, 2020, by the Russian Ministry of Health as Gam-Covid-Vak. On February 2, 2021, a preliminary analysis of the human phase 3 trials of this vaccine was published, showing an effect of 91.6% without unusual side effects. Sputnik V is an adenovirus carrier vaccine. Generally, vaccines with adenovirus vector have been extensively studied and have shown a good cellular and humoral immune response after the first injection. Injection of the second dose of the vaccine has shown stronger immunity and longer duration^2–4^. To ensure lasting safety, Russian scientists, using two types of adenovirus vectors (rAd26 and rAd5) for the first and second vaccinations, came up with an important idea to boost the effectiveness of the vaccine, which successfully completed the first and second clinical trial phases. The vaccine is stored and distributed at − 18 °C, but storage at 2–8 °C, has a favorable temperature profile for global distribution^3^. As of February 2021, 21 countries have authorized Sputnik V emergency use and more than one billion doses of the vaccine have been ordered for immediate worldwide distribution. Although based on preliminary results published in the clinical stages of the vaccine trial, Sputnik V manufacturers claim that the vaccine is highly immunogenic and has no side effects, it is necessary to evaluate the possible side effects and measure the immunogenicity of this vaccine. The aim of this study is to determine the humoral immunogenicity and possible side effects of the Sputnik V vaccine in the health care workers of Mazandaran University of Medical Sciences as the first group to receiving COVID-19 vaccines in Iran. This study will provide reassurance information regarding what vaccine recipients might expect after vaccination.

## Material and methods

### Study design, participants and evaluation of vaccine side effects

**Figure 1:**
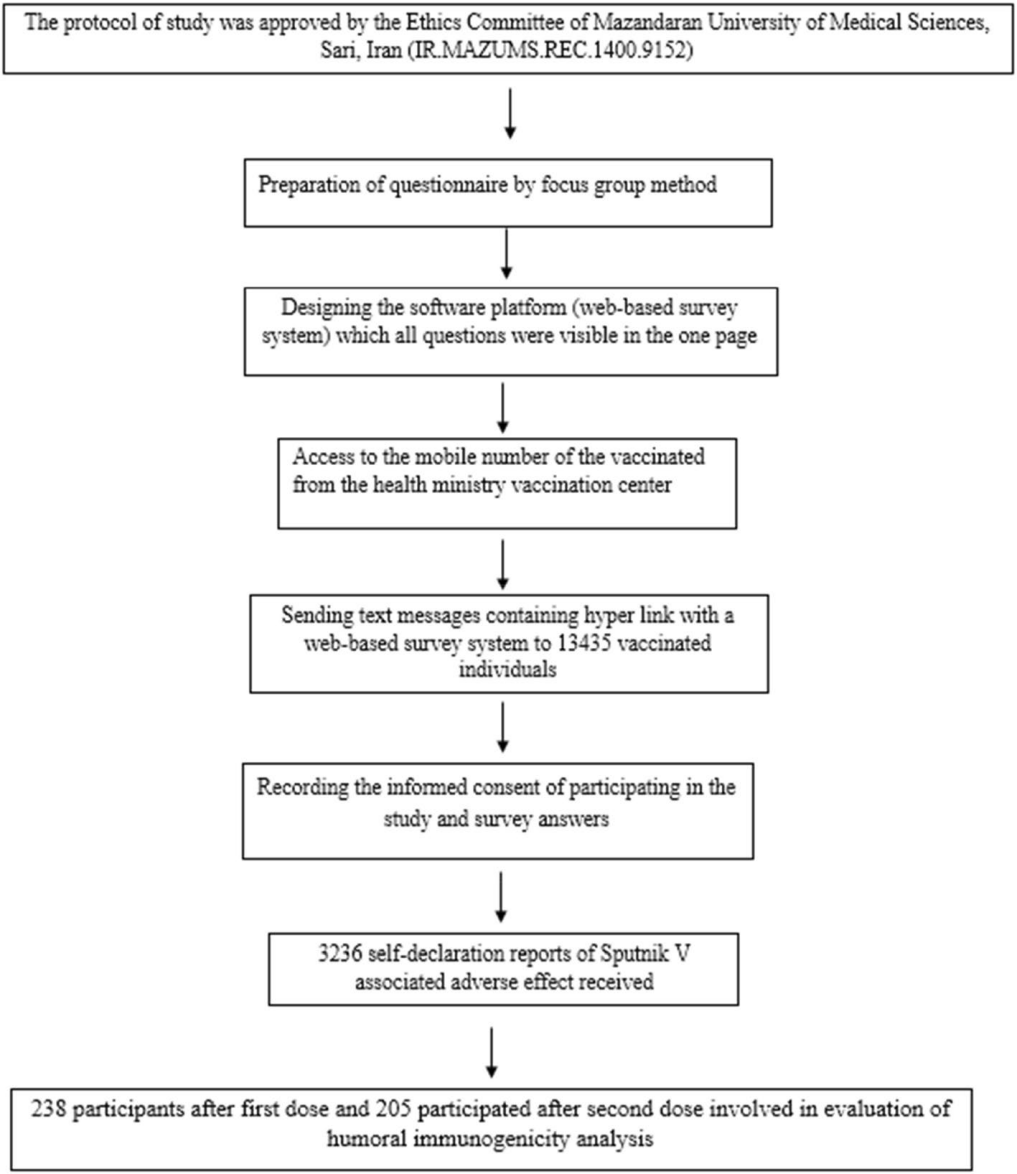
Flow chart of study design.

This observational (survey-based) and cohort study was performed at the Mazandaran University of Medical Sciences, between February 2021 and April 2021. The Strengthening the Reporting of Observational Studies in Epidemiology guideline (STROBE) was followed^5^. The study was approved by the Ethics Committee of Mazandaran University of Medical Sciences (Code: IR. MAZUMS.REC.1400.9152).The study was conducted according to the criteria set by the declaration of Helsinki. Moreover, written informed consent was obtained from all subjects or, for subjects under the age of 18, from a parent and/or legal guardian. In this study, all methods were carried out in accordance with the relevant guidelines and regulations. The study design is illustrated in Fig. 1.

Inclusion criteria were healthcare workers in Mazandaran province who were vaccinated with the Sputnik V COVID-19 vaccine during the first stage of COVID-19 vaccination in Iran, while the individuals who were vaccinated in March 2021 by Covaxin or Sinopharm COVID-19 vaccine were excluded from this report. To evaluate the side effects of the Sputnik V vaccine, the sample size was determined by the census method, therefore all health care workers who were affiliated to Mazandaran University of Medical Sciences and were vaccinated by the Sputnik V vaccine were included in the study. The self-declaration questionnaire of COVID-19 vaccine side effects was written and reviewed by a panel of six infectious diseases specialists and two epidemiologists to assess its content validity. Also, the reliability of the questionnaire was assessed by a group of lately vaccinated health care staff. The vaccine side effects questionnaire was available to vaccine recipients via text messages from smartphones linked to a free web-based survey system within 8 days after vaccination and their self-declaration responses were recorded. The questionnaire included twenty-nine multiple-choice items such as demographic data, the past COVID-19 infection, vaccination date and the number of doses, vaccine side effects including local in injection site side effects and systemic side effects, the onset and duration of side effects and other vaccinations and one question with the possibility of descriptive answers about side effects that were not mentioned in the questionnaire.

### Humoral immunogenicity analysis by detection of SARS-CoV-2 RBD antibody and SARS-CoV-2 neutralizing antibody

For the evaluation of humoral immune response, serum samples of all high-risk occupational groups vaccinated by Sputnik V in the Razi teaching hospital (referral center of infectious disease in Mazandaran province) were analyzed for the detection of specific antibodies of the receptor-binding domain of SARS-CoV-2 glycoprotein S (SARS-CoV-2 RBD antibody) and SARS-CoV-2 neutralizing antibody by Enzyme-linked immunosorbent assay (ELISA). The SARS-CoV-2 RBD antibody and SARS-CoV-2 neutralizing antibody ELISA kits (Pishtaz Teb, Tehran, Iran) were used according to the manufacturer’s instructions. The titer of IgG to RBD of SARS-CoV-2 virus was measured on days 21 and day 42 after the first dose vaccination (21 days after first and second doses the serum sample was harvested). To detect SARS-CoV-2 neutralizing antibodies, the competitive method based on quantitative inhibition of RBD and ACE2 binding, which has the ability to identify all classes of human antibodies that neutralize SARS-CoV-2 virus performed and the human neutralizing antibody was used as the standard. The titer of neutralizing antibodies was measured on day 42 after the first dose vaccination. The manufacturer-reported sensitivity and specificity were respectively, 97.1% and 100% for the SARS-CoV-2 Anti-RBD IgG kit and the accuracy of the SARS-CoV-2 Neutralizing Ab kit was 99.52%. In the serum samples of normal individuals, the mean and three folds of the standard deviation was the basis for determining cut-off, according to the kit instructions. The cut-off values were calculated 2.5 μg/mL (negative result < 2.5 μg/mL ≤ positive result) for SARS-CoV-2 neutralizing antibody and 5 μg/mL (negative result < 5 μg/mL ≤ positive) result for SARS-CoV-2 RBD antibody^6^.

### Statistical analysis

Data were analyzed using the SPSS package (version 24.0; Windows). Descriptive analyses used frequency and percentage based on the non-missing sample size. Differences between groups were determined by the chi-square test or Fisher's exact test with P < 0.05 considered statistically significant.

## Results

During February–April 2021, a total of 13,435 healthcare workers affiliated to Mazandaran University of medical sciences vaccinated by Sputnik V COVID-19 vaccine. We received 3236 self-declaration reports of Sputnik V associated adverse events during this period. The mean age of vaccine recipients who submitted for adverse events were 39.32 ± 10.19, and it ranged between 19 and 78 years old with a median of 38 years old; 38.8% were men and 61.2% were women (Table1).

**Table 1:** Demographic features of participants reported side effect after first and second dose vaccination by Sputnik V COVID-19 vaccine.

Demographic features of participants after first and second dose of vaccine	Gender		Total
	Female	Male	
**First dose**			
Number of participants	1364	830	2194
Side effects (%)	62.2%	37.8%	100.0%
Among gender groups (%)	68.8%	66.2%	67.8%
Total (%)	42.2%	25.6%	67.8%
**Second dose**			
Number of participants	618	424	1042
Side effects (%)	59.3%	40.7%	100.0%
Among gender groups (%)	31.2%	33.8%	32.2%
Total (%)	19.1%	13.1%	32.2%
**Total**			
Total number of participants	1982	1254	3236
Total reported side effects (%)	61.2%	38.8%	100.0%
**Age**			
Mean ± SD	37.77 ± 9.395	41.75 ± 10.886	39.32 ± 10.19
Minimum–maximum	19–70	21–78	19–78

**Figure 2:**
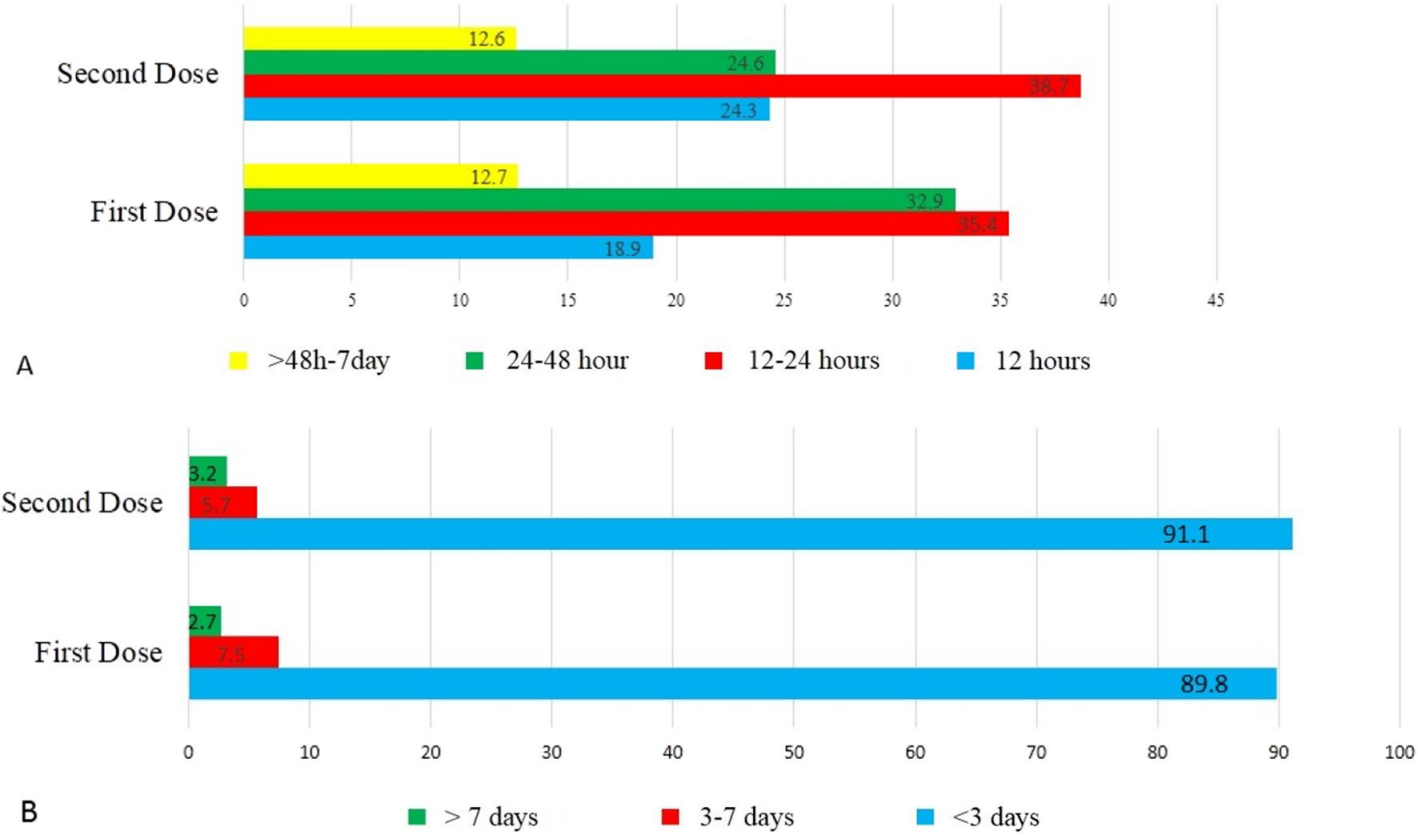
The onset (**A**) and duration (**B**) of side effects after first and second dose of Sputnik V COVID-19 vaccine. The numbers in the bars are in percentage.

The local and systemic side effect reported within 8 days after injection of the first and the second dose of the Sputnik V vaccine is shown in Table 2. The most common side effect was a pain in the injection site (56.9%), fatigue (50.9%), body pain (43.9%), headache (35.7%), fever (32.9%), joint pain (30.3%), chilling (29.8%) and drowsiness (20.3%) respectively (Table2). The less common vaccine side effects were diarrhea (5%), depression (6.1%), rash (2.3%), vomiting (1.3%), constipation (0.1%), anaphylaxis shock (0.1%) and vasovagal syncope (0.60%) respectively. Although rarely reported side effects included increased heart rate, itching all over the body, shortness of breath, the dry and bad taste of mouth, temporary hair loss, runny nose and sore throat were reported in less than 0.06% of participants. Most vaccine side effects such as pain in the injection site, fever, chilling, headache, dizzying, body pain, fatigue, weakness, nausea, joint pain and drowsiness significantly decreased in the second dose in comparison with the first dose (Table 2). Generally, 216 (6.67%) of participants were COVID-19 positive in the last 3 months before vaccination and 740 (22.86%) of participants had at least one-time COVID-19 infection more than 3 months and less than a year before vaccination. The most common side effects of Sputnik V COVID-19 vaccine based on demographic features and past COVID-19 is shown in the Table 3. Given the median age of this study’s participants was “38 years old”, this age had been used as a cut-off to present of the Sputnik V COVID-19 vaccine side effects in participants. Most common side effects were significantly more observed among female and participants younger than 38 years old (P < 0.05). In the other hand participants with previous COVID-19 infection experienced a significantly higher rate of pain in the injection site and some systemic side effects such as body pain, fatigue, and weakness in comparison with participants without previous COVID-19 infection, however, some systemic side effect such as fever, headache, and joint pain were significantly less common in the participants with previous COVID-19 infection (P < 0.05) (Table 3). The onset and duration of vaccine side effects after the first and second dose is illustrated in Fig. 2. The onset of side effects in the participants were 12–24 h (35.4–38.7%), 24–48 h (32.9–24.6%), 12 h (18.9–24.3%), and more than 48 h to 7 days after vaccination (12.7–12.6%) respectively while the duration of experiencing vaccine side effects was less than 3 days in most participants (89.8–91.1%). After receiving the first dose and the second dose of vaccine 8 (0.8%) of participants self-declared to infected by COVID-19 and they claimed their COVID-19 infection had confirmed by real-time reverse transcriptase-PCR (RT-PCR) assay, CT-Scan or rapid lateral flow test.

**Table 2:** The side effects of Sputnik V COVID-19 vaccine after first and second dose.

	First dose (N = 2194) N (%)	Second dose (N = 1042) N (%)	Total (N = 3236) N (%)	P value
**Local side effects on the injection site**				
Pain	1278 (58.2)	564 (54.1)	1842 (56.9)	**0.028**
Swelling	138 (6.3)	89 (8.5)	227 (7)	**0.022**
Redness	125 (5.7)	68 (6.5)	193 (6)	0.382
**Systemic side effects**				
Fever	801 (36.5)	248 (23.8)	1049 (32.4)	** < 0.0001**
Chilling	727 (33.1)	237 (22.7)	964 (29.8)	** < 0.0001**
Headache	835 (38.1)	321 (30.8)	1156 (35.7)	** < 0.0001**
Dizzying	318 (14.5)	129(12.4)	447 (13.8)	0.114
Body pain	1067 (48.6)	396 (38)	1463 (45.2)	** < 0.0001**
Fatigue	1189 (54.2)	459 (44)	1648 (50.9)	** < 0.0001**
Weakness	1023 (46.6)	397 (38.1)	1420 (43.9)	** < 0.0001**
Nausea	201 (9.2)	68 (6.5)	296 (8.3)	**0.012**
Vomiting	30 (1.4)	13 (1.2)	43 (1.3)	0.870
Diarrhea	113 (5.2)	50 (4.8)	163 (5)	0.731
Constipation	1 (0.04)	2 (0.2)	3 (0.1)	0.244
Joint pain	707 (32.2)	275 (26.4)	982 (30.3)	**0.001**
Vasovagal syncope	15 (0.7)	6 (0.6)	21 (0.60)	0.818
Anaphylaxis shock	4 (0.2)	0	4 (0.1)	0.312
Rash	47 (2.1)	26 (2.5)	73 (2.3)	0.528
Depression	143 (6.5)	54 (5.2)	197 (6.1)	0.157
Drowsiness	470 (21.4)	187 (17.9)	657 (20.3)	**0.022**

P value < 0.05 showed by boldface.

**Table 3:** The most common side effects of Sputnik V COVID-19 vaccine based on demographic features and past COVID-19.

	Female (N = 1982) N (%)	Male (N = 1252) N (%)	P value	≤ 38 years old (N = 1518) N (%)	> 38 years old (N = 1718) N (%)	P value	COVID-19 in the last 3 months (N = 216) N (%)	P value	COVID-19 more than 3 months and less than a year before (N = 740) N (%)	P value
Pain in injection site	1240 (62.6)	602 (48)	** < 0.0001**	931 (61.3)	911(53)	** < 0.0001**	140 (64.8)	**0.016**	468 (63.2)	** < 0.0001**
Fever	676 (34.1)	373 (29.7)	**0.010**	565 (37.2)	484 (28.2)	** < 0.0001**	84 (38.9)	**0.042**	275(37.2)	**0.002**
Chilling	669 (33.8)	295 (23.5)	** < 0.0001**	528 (34.8)	436 (25.4)	** < 0.0001**	67 (31)	0.700	248(25.7)	**0.013**
Headache	768 (38.8)	387 (30.9)	** < 0.0001**	574(37.8)	582 (33.9)	**0.021**	101(46.8)	**0.001**	320(43.2)	** < 0.0001**
Body pain	957 (48.3)	506 (40.4)	** < 0.0001**	747 (49.2)	716 (41.7)	** < 0.0001**	116(53.7)	**0.001**	392 (53)	** < 0.0001**
Fatigue	1083 (54.6)	565 (45.1)	** < 0.0001**	841 (55.4)	807 (47)	** < 0.0001**	132(61.1)	**0.002**	426 (57.6)	** < 0.0001**
Weakness	951 (48)	469 (37.4)	** < 0.0001**	729(48)	691 (40.2)	** < 0.0001**	115(53.2)	**0.004**	378 (51.1)	** < 0.0001**
Joint pain	648 (32.7)	334 (26.6)	** < 0.0001**	482 (31.8)	500 (29.1)	0.108	78(36.1)	**0.044**	294(39.7)	** < 0.0001**
Drowsiness	435 (21.9)	222 (17.7)	**0.004**	360 (23. 7)	297 (17.3)	** < 0.0001**	52(24.1)	0.161	174 (23.5)	0.114

P value < 0.05 showed by boldface.

### The vaccine side effects and the immunogenicity of the Sputnik V COVID-19 vaccine among high-risk occupational groups in the Razi teaching hospital

Participants in the Razi teaching hospital with average age 37.91 ± 9.39 years and range (21–71 years) including 152 (63.9%) female and 86 (36.1%) male evaluated for immunogenicity assays. By day 21 following the first dose vaccination, SARS-CoV-2 RBD antibodies were observed in 219(92%) of vaccine recipients, comparing undetectable titer in 19 (8%) participants. By day 21 after the second dose vaccination (day 42 after the first dose vaccination), SARS-CoV-2 RBD antibodies were observed in 200 (97.6%) of vaccine recipients, comparing undetectable titer in 5 (2.4%) participants. The SARS-CoV-2 neutralizing antibody was observed in 187 (91.2%) vaccine recipients, comparing undetectable titer in 18 (8.8%) participants. Table 4 is shown the potential variables affecting the antibody production and common systemic side effects in participants based on detection of SARS-CoV-2 RBD and SARS-CoV-2 neutralizing antibodies. While the production of SARS-CoV-2 RBD antibody after the first dose and SARS-CoV-2 neutralizing antibody were significantly more common in the participant with past COVID-19 infection, the other potential variables such as demographic features or other viral vaccinations were not associated with antibody production (Table 4). In the other hand the occurrence of vaccine side effects was not related to antibody production in participants. The COVID-19 infection was confirmed in two participants in the Razi teaching hospital after receiving the second dose of the vaccine. These individuals had symptoms of COVID-19 and the real-time reverse transcriptase-PCR (RT-PCR) assay for severe acute respiratory syndrome coronavirus-2 (SARS-CoV-2) of nasopharyngeal swab got positive for Alfa (B.1.1.7) SARS-CoV-2 variant after days 7 and 8 after receiving dose 2 vaccines. The SARS-CoV-2 RBD antibody after the first and second dose also the SARS-CoV-2 neutralizing antibody after the second dose were detectable in both participants.

**Table 4:** Potential variables affecting the antibody production and common systemic side effects based on detection of SARS-CoV-2 RBD and SARS-CoV-2 neutralizing antibody.

Potential variables affecting the antibody production and common systemic side effects	SARS-CoV-2 RBD antibody positive after dose 1 (N = 219) N (%)	SARS-CoV-2 RBD antibody negative after dose 1 (N = 19) N (%)	P value	SARS-CoV-2 RBD antibody positive after dose 2 (N = 200) N (%)	SARS-CoV-2 RBD antibody negative after dose 2 (N = 5) N (%)	P value	SARS-CoV-2 neutralizing antibody positive after dose 2 (N = 186) N (%)	SARS-CoV-2 neutralizing antibody negative after dose 2 (N = 18) N (%)	P value
**Demographic features and history of COVID-19**									
Female	142 (64.86)	10 (52.6)	0.324	128 (64)	3 (60)	1	119 (64)	11 (61.1)	0.802
Male	77 (35.2)	9 (47.4)		72 (36)	2(40)		67 (36)	7 (38.9)	
≤ 38 years old	119 (54.3)	10 (52.6)	1	107 (53.5)	2(40)	0.667	100 (53.8)	9 (50)	0.808
> 38 years old	100 (45.7)	9 (47.4)		93 (46.5)	3 (60)		86 (46.2)	9 (50)	
*Past COVID-19 infection	56 (25.6)	1 (5.3)	**0.04**	51 (25.5)	0	0.335	50 (26.9)	0	**0.008**
**Common systemic side effects**									
Fever	57 (26)	4 (21)	0.788	34 (17)	0	0.593	31 (16.7)	3 (16.7)	1
Chilling	51 (23.3)	7 (36.8)	0.262	35 (17.5)	0	0.591	33 (17.2)	2 (11.2)	0.744
Headache	63 (28.8)	6 (31.1)	0.795	50 (25)	2 (40)	0.603	46 (24.7)	6 (33.3)	0.4.8
Body pain	89 (40.6)	8 (42.1)	1	62 (31)	0	0.325	59 (31)	3 (16.7)	0.283
Fatigue	95 (43.4)	8 (42.1)	1	72 (36)	0	0.164	66 (35.5)	6 (33.3)	1
Weakness	71 (32.4)	7 (36.8)	0.800	62 (31)	1 (20)	1	57 (30.6)	6 (33.3)	0.794
Joint pain	56 (25.6)	1 (5.3)	0.051	40 (20)	0	0.585	37 (19.9)	3 (16.7)	1
Other vaccinations**									
Hepatitis B vaccine	5 (2.3)	0	1	4 (2)	0	1	4 (2.2)	0	1
Influenza vaccine	5 (2.3)	1 (5.3)	0.396	6(3)	0	1	6 (3.2)	0	1

P value < 0.05 showed by boldface; *previous COVID-19 within a last year; **received vaccine in the last 3 months before COVID-19 vaccination.

## Discussion

In this study in Iran, we have investigated side effects and immunogenicity following the administration of the Sputnik V COVID-19 vaccine in healthcare workers. The first evidence of safety and efficacy of the Sputnik V COVID-19 vaccine was obtained from a randomized controlled phase 3 trial by Logunov et al. at 25 hospitals and polyclinics in Moscow, Russia and 21 977 adults were randomly assigned to the vaccine group (n = 16,501) or the placebo group (n = 5476). The interim results of this trial showed that the vaccine’s efficacy was 91.6%, without serious side effects^3^. The most common side effects of the phase 3 trial of the Sputnik V vaccine were flu-like illness, injection site reactions, headache, and asthenia. The most common vaccine side effects in our studied population included pain at the injection site, fatigue, body pain and headache were similar to the most common side effects reported after injection of Pfizer-BioNTech and the Oxford-AstraZeneca^7,8^. Pagotto et al. reported side effects followed vaccination by Sputnik V vaccine of 683 healthcare workers in Argentina which (57%) of the participants reported pain at the injection site, and (11%) had redness and swelling. The most common systemic side effects were new or worsened muscle pain (58%), fever (40%), headache (33%) and diarrhea (5%)^9^.The incidence of local and systemic side effect of Pagotto et al. study was similar to our findings. Although Montalti et al. reported participants experiencing both local and systemic adverse effect increased in the second dose of the Sputnik V vaccine when compared to the first dose^10^. In our study, the vaccine side effects significantly decrease after the second dose in comparison with the first dose (Table 3). In contrary to our results, Menin et al. in a large prospective observational study in the UK found that systemic side effects after receiving Pfizer-BioNTech vaccine increased in the second dose^8^. The differences between these findings may be explained by the nature and immunogenicity of these vaccines. Similar to observational results on side effects of Pfizer-BioNTech, the Oxford-AstraZeneca vaccine and Sputnik V vaccine, we found in our study that Sputnik V side-effects were significantly more prevalent in female than in male, in younger participants than older individuals^7–9^. Moreover, Menin et al. reported local side effect in the injection site (1.4 times after the first dose of Oxford-AstraZeneca and 1.2 times after the first dose of Pfizer-BioNTech) and systemic side effects (1.6 times after the first dose of Oxford-AstraZeneca and 2.9 times after the first dose of Pfizer-BioNTech) were higher in individuals previously infected than in those without known past COVID-19 infection^8^. Strikingly, in our study participants with past COVID-19 infection experienced the same trend of side effects of other studies, as well as a significantly higher rate of some systemic side effects including body pain, fatigue, and weakness in comparison with participants without past COVID-19 infection, however, some systemic side effect such as fever, headache, and joint pain were significantly less common in the participants with past COVID-19 infection^7,8^. Although, Logunov et al. in the interim results of the phase 3 trial of the Sputnik V vaccine reported that no serious adverse events were reported during the study, in our study the incidence of self-reports serious side effects such as anaphylaxis shock was about 0.1%. Anaphylaxis is a life-threatening allergic reaction that does occur after vaccination. The centers for disease control and prevention (CDC) has reported 21 cases of anaphylaxis after reported administration of 1,893,360 first doses of Pfizer-BioNTech COVID-19 vaccine^9^. Due to the observational nature of our study, we couldn’t follow the participants with these serious side effects. Further comparative clinical studies are needed to estimate the rate of rare side effects of COVID-19 Sputnik V vaccines.

In our study after receiving the first dose of vaccine, 18 (0.8%) and after receiving the second dose of vaccine 8 (0.8%) of participants reported to be infected by COVID-19. Although our participants were not tested to be COVID-19 negative before vaccination, the incidence of COVID-19 after the first and second dose of vaccine was a much lower rate than the interim results of phase 3 clinical trials of the Sputnik V vaccine^3^. We performed the humoral immunogenicity analysis after vaccination among high-risk occupational participants in the Razi teaching hospital. Ninety eight percent of participants in the vaccine group of phases 3 of a clinical trial of the Sputnik vaccine had a detectable level of RBD-specific IgG and a seroconversion rate of 98.25%. Although we did not test the level of antibody before vaccination to evaluate the seroconversion rate, we found that more than 90% of individuals after the first and second dose of vaccine had a detectable level of SARS-CoV-2 RBD antibody and SARS-CoV-2 neutralizing antibody and notably the frequency of individual with detectable antibody was higher after the second dose (97.6%) in comparison with the first dose (92%). However, cellular immunogenicity evaluations and long-term surveillance for evaluation of the antibody titer are recommended to ensure protection persistence by the COVID-19 vaccine or probably needing booster dose of vaccine. Some studies reported vaccines could increase immunogenicity in individuals with past infection. Inconsistent with these reports, in the analysis of potential variables affecting the antibody production and common systemic side effects based on detection of SARS-CoV-2 RBD and SARS-CoV-2 neutralizing antibody, we found that the frequency of participants with detectable and SARS-CoV-2 antibody is significantly more in than individuals with without evidence of past COVID-19 infection in comparison with individuals without past evidence if COVID-19 infection^11,12^. Our study has some drawbacks. The first drawback of this study is due to the survey-based strategy that may lead to self-selection bias when perhaps only the highly motivated participants filled in the. However, all the studied populations were healthcare workers who have a high level of medical expertise, the self-reporting nature of the collected data, can make information bias and can jeopardize its reliability during clinical evaluation. To the best of our knowledge, this study was the first large sample size observational study dealing with the Sputnik V vaccine side effects, and the first designed study evaluating the vaccine side effects among the Iranian population. In conclusion, side effects of the Sputnik V vaccine are more frequent in female and younger individuals and these adverse effects are frequently started within 24 h after vaccination and will resolve in less than 3 days. Moreover, our findings support interim results from randomized controlled trials showing evidence of high humoral immunogenicity against COVID-19 after receiving the Sputnik V vaccine.
